# TRIM E3 Ligases Interfere with Early and Late Stages of the Retroviral Life Cycle

**DOI:** 10.1371/journal.ppat.0040016

**Published:** 2008-02-01

**Authors:** Pradeep D Uchil, Brian D Quinlan, Wai-Tsing Chan, Joseph M Luna, Walther Mothes

**Affiliations:** Section of Microbial Pathogenesis, Yale University School of Medicine, New Haven, Connecticut, United States of America; Institute for Research in Biomedicine, Switzerland

## Abstract

Members of the TRIpartite interaction Motif (TRIM) family of E3 ligases have been shown to exhibit antiviral activities. Here we report a near comprehensive screen for antiretroviral activities of 55 TRIM proteins (36 human, 19 mouse). We identified ∼20 TRIM proteins that, when transiently expressed in HEK293 cells, affect the entry or release of human immunodeficiency virus 1 (HIV), murine leukemia virus (MLV), or avian leukosis virus (ALV). While TRIM11 and 31 inhibited HIV entry, TRIM11 enhanced N-MLV entry by interfering with Ref1 restriction. Strikingly, many TRIM proteins affected late stages of the viral life cycle. Gene silencing of endogenously expressed TRIM 25, 31, and 62 inhibited viral release indicating that they play an important role at late stages of the viral life cycle. In contrast, downregulation of TRIM11 and 15 enhanced virus release suggesting that these proteins contribute to the endogenous restriction of retroviruses in cells.

## Author Summary

A lot of excitement in the field of innate immunity to retroviruses such as HIV has come from the discovery of TRIM5 as a key player in cross species restriction. TRIM5 belongs to a family of E3 ligases with over 70 members, a number of which have exhibited antiviral activity. These findings have led to the hypothesis that several TRIM proteins may contribute to the innate immunity to retroviruses. In this manuscript, we systematically test the antiviral activities of 55 human and mouse TRIM proteins. The results are astonishingly complex with activities affecting both early and late stages of the retroviral life cycle. Importantly, a number of TRIM proteins that affect HIV or MLV replication upon overexpression, enhance virus entry or release when downregulated by gene silencing. These experiments suggest that additional TRIM proteins contribute to the endogenous restriction of retroviruses. Future work should focus on the identification of TRIM proteins that are upregulated specifically in response to interferons as well as the mechanisms by which the identified proteins interfere with retroviral replication.

## Introduction

Host cells express specific proteins to interfere with the replication of retroviruses. These proteins are referred to as restriction factors and are considered to be a part of an innate or intrinsic immune system [1–5]. The interferon inducible cytidine deaminase APOBEC3G is packaged into retroviruses and exerts its antiviral effect during reverse transcription. TRIM5 and murine Fv1 belong to a class of restriction factors that interfere with virus replication before and after reverse transcription, respectively. The *Fv1* gene encodes an endogenous retroviral Gag found in the mouse genome and has two main alleles [6]. *Fv1^n^*, found in NIH Swiss mice, restricts infection by B-tropic MLV (B-MLV) but not N-tropic MLV (N-MLV). In contrast, *Fv1^b^*, found in BALB/c mice, restricts N-MLV and not B-MLV [7]. NB-tropic MLV replicates in both mouse strains. The residues critical for the N-B tropism of MLV map to the retroviral capsid protein. TRIM5 was identified as a protein responsible for the species-specific restriction of HIV entry [8,9]. Moreover, TRIM5 also mediates the Ref1 restriction of specific mouse retroviruses such as N-MLV in mammalian cells [10–12]. TRIM5 binds to incoming retroviral capsids via its C-terminal B30.2 or the SPRY (SPla/RYanodine receptor) domain causing premature capsid disassembly [13–15].

TRIM5 belongs to the large family of TRIM/RBCC proteins with over 70 members. TRIM proteins display elements of a conserved modular tripartite motif structure consisting of an N-terminal E3 ubiquitin ligase RING (Really Interesting New Gene) domain followed by one or two zinc binding motifs named B-box and a predicted coiled coil (CC) region (see Table 1). The C-terminus is highly variable and contains specific domains such as the B30.2/PRY-SPRY domain (Table 1). The presence of a RING domain suggests that these proteins function as E3 ubiquitin ligases. The associated B-box and coiled coil are believed to participate in protein-protein interactions and formation of macromolecular complexes [16,17].

**Table 1:**
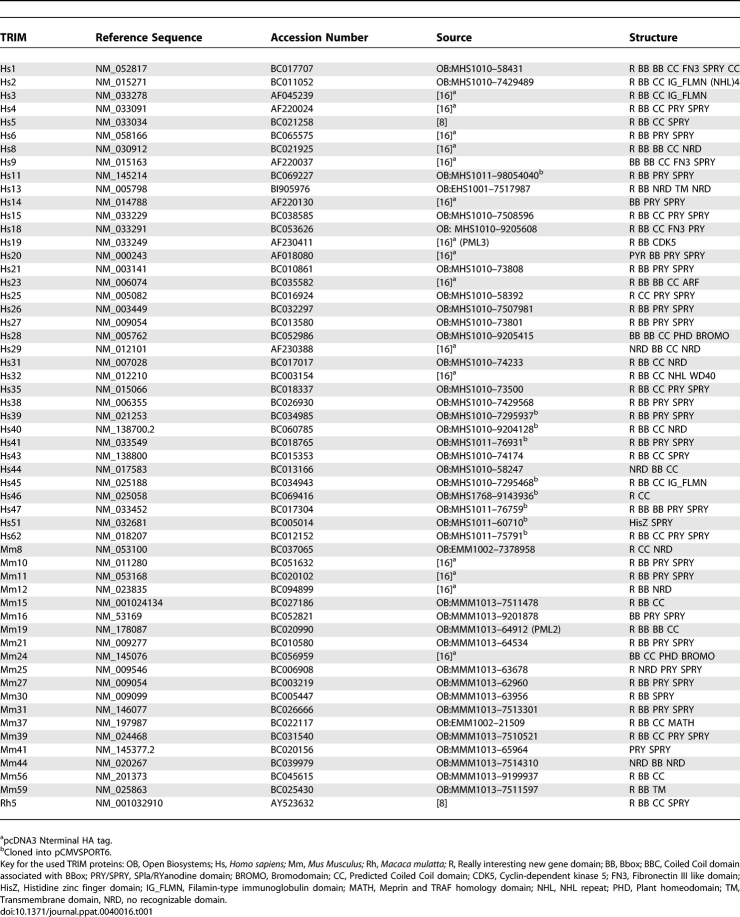
TRIM Proteins Included in the Study

TRIM proteins localize to various regions within the cells and many define specific nuclear (TRIM19/PML) [18] or cytoplasmic compartments (TRIM5) [8,19]. Others such as TRIM1, 9 and 18 have been shown to associate with microtubules [20–23]. Proposed physiological roles for TRIM proteins include fundamental cellular processes such as apoptosis, transcription, differentiation, and regulation of cell cycle progression [17]. Moreover, mutations in several TRIM proteins have been linked to human disease [17].

A number of TRIM proteins besides TRIM5 and its close relatives have been shown to possess antiviral activities [2,3,17,24]. For example, TRIM1 has been shown to restrict N-MLV [12]. Broad antiviral activities have been described for TRIM19, the defining component of PML bodies in the nucleus. The list of viruses inhibited by TRIM19 includes vesicular stomatitis virus, influenza A virus, human cytomegalovirus, herpes simplex type 1, Ebola virus, Lassa fever virus, lymphocytic choriomeningitis virus, human foamy virus and HIV [2,18]. TRIM28 restricts MLV in cells of germline origin by inhibiting LTR-driven transcription [24]. TRIM22 and TRIM32 were reported to attenuate transcription of the HIV LTR [25,26]. The identification of TRIM25 as a K63 specific ubiquitin E3 ligase activating RIG-I presents direct evidence that TRIM proteins regulate innate immunity to viral infection [27]. The recognition and suppression of Sendai virus, New Castle disease virus and vesicular stomatis virus (VSV) replication by RIG-I depends on functional TRIM25 [27]. Thus, the association of several family members with antiviral activities coupled with the fact that many of them are induced by interferons [2] has led to the hypothesis that members of TRIM family proteins are a part of innate immune system to counter intracellular pathogens [2,17,28].

To systematically test antiretroviral activities of TRIM proteins, we investigated the ability of 55 TRIM proteins (36 human, 19 mouse) to interfere with early and/or late stages of the retroviral life cycle.

## Results

### TRIM Proteins Inhibiting or Enhancing Retroviral Entry

We screened for potential antiviral activities of 36 human and 19 mouse TRIM proteins (Table 1) by transient expression in HEK293 cells. These cells are highly permissive for most retroviruses and are easily transfectable. We first performed control experiments to verify that the transfection of 50 ng plasmids encoding TRIM proteins in a 24-well format minimally induced apoptosis and had little effect on cell viability or gene expression (Figure S1A–S1C). In the case of human and mouse TRIM11, transfected DNA levels were reduced to 10 ng (Figure S1C).

**Figure 1:**
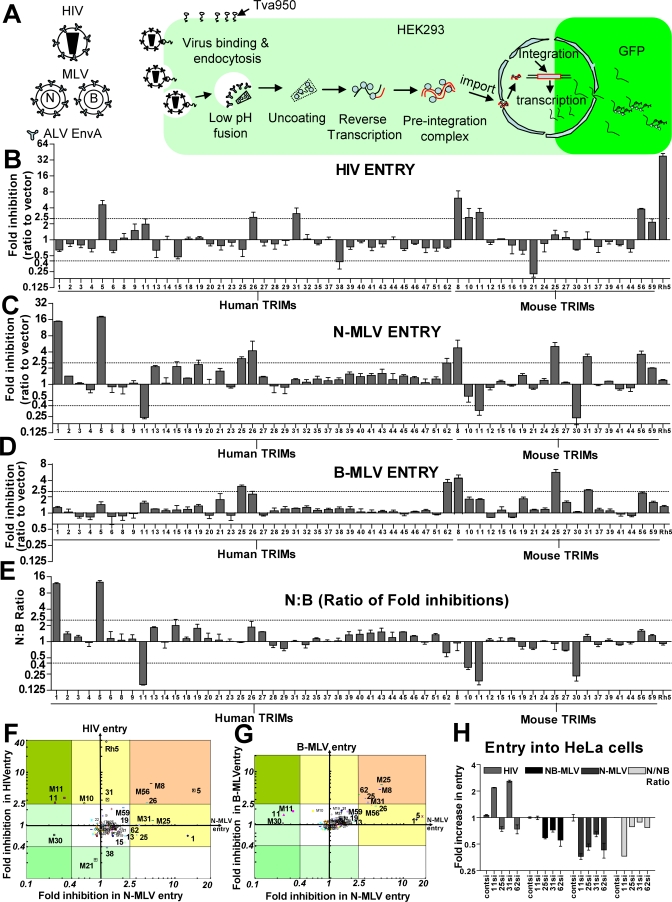
TRIM Proteins Inhibiting or Enhancing Retroviral Entry (A) Experimental design: HIV, N, and B-MLV viruses carried an ALV-A envelope and the target HEK293 cells expressed cognate receptor Tva950 and TRIM proteins. Effects on entry were measured at the level of gene expression using GFP as reporter. (B–D) Effects of TRIM protein expression on the entry of HIV (B), N-MLV (C), and B-MLV (D) shown as fold inhibition using a log2 scale with standard errors. The dotted line represents the statistically determined cut-off value. (E) Ratio of fold inhibition in N- and B-tropic MLV entry. (F, G) Scatter plot comparing the effects on entry for one virus against another as indicated. (H) Effect of silencing endogenous TRIM proteins 11, 25, 31, and 62 in HeLa cells using specific or control siRNA on HIV, NB- and N-MLV entry. The ratio of fold increase in entry of N versus NB-MLV is shown to the right.

We then analysed the ability of the TRIM proteins to interfere with viral entry defined here as all early events in the retroviral life cycle leading up to the establishment of viral gene expression (Figure 1A). To identify activities directed specifically against incoming retroviral capsids, all viruses carried the same subgroup A envelope glycoprotein of ALV (ALV-A) and target cells expressed the cognate receptor Tva950 [29]. To guarantee a ∼90% probability of Tva950 co-expression with each TRIM protein, plasmids encoding for both proteins were co-transfected 36 h prior to initiating infection. Infection levels were determined by measuring the expression of cytoplamic GFP from integrated viral genomes.

To perform an entry screen for HIV, reporter viruses were generated in HEK293 cells by transfecting HXB2ΔEnv-GFP, an HXB2 derivative lacking envelope and encoding GFP instead of the nef gene, together with a plasmid encoding ALV-A Env. Culture supernatants were harvested 48 h later and tested for their ability to infect HEK293 cells expressing Tva950 in the presence or absence of individual TRIM protein. To evaluate the significance of the observed inhibitory and enhancing effects standard statistical analysis was employed to arrive at a cut-off of 2.5 (by adding the maximum variability between control samples and two times the standard deviations). HIV entry was potently blocked by rhesus TRIM5 (38-fold) and to a lesser extent by human TRIM5 (5-fold) confirming previous results [8,10–12] (Figure 1B). Interestingly, no other TRIM protein affected HIV entry as potently as rhesus TRIM5. Mouse TRIM8 inhibited HIV entry about 6-fold. Moderate inhibitory effects were observed for human TRIM11, 26, 31 and mouse 10, 11 and 56. Expression of human TRIM38 and mouse TRIM21 enhanced HIV entry.

To perform a similar experiment for N-tropic MLV (N-MLV), reporter viruses were generated in HEK293 cells by transfecting plasmids encoding for N-tropic MLV GagPol, MLV LTR-GFP and ALV-A Env. Viruses were harvested as above and the susceptibility of HEK293 cells expressing Tva receptor and individual TRIM proteins to N-MLV was tested. N-MLV was strongly inhibited by TRIM1 (15-fold) and TRIM5 (18-fold) (Figure 1C). A number of additional TRIM proteins moderately affected N-MLV entry (human TRIM25, 26 and 62; mouse TRIM8, 25, 31 and 56). Interestingly, human and mouse TRIM11 as well as mouse TRIM30 enhanced N-MLV entry (4.5-, 3- and 4.5-fold, respectively).

The inhibitory pattern observed for N-MLV was largely distinct from HIV (Figure 1B and 1C). Notable exceptions included human TRIM26, mouse TRIM8 and 56 that affected both HIV and MLV. Opposite effects were observed for the TRIM11 proteins. While they inhibited HIV entry, enhancing effects were observed for MLV. TRIM proteins specifically affecting N-MLV were human and mouse TRIM25, human TRIM62, mouse TRIM31 and mouse TRIM30. In contrast, human TRIM proteins 31, 38 and the mouse proteins 10 and 21 specifically affected HIV entry. A scatter plot depicting fold inhibition in infectivity for HIV versus N-MLV summarizes these results (Figure 1F).

The interference of TRIM 1 and 5 with MLV entry is specific for capsid determinants of N-MLV but not of B-MLV [10–12]. To test which TRIM proteins are specific for N-MLV, we performed an identical entry experiment for B-MLV. As previously reported, human TRIM1 and TRIM5 exhibited no effect on B-MLV entry (Figure 1D). In contrast, the remaining TRIM proteins exhibited an inhibitory profile that resembled that observed for N-MLV (compare Figure 1D to 1C). Interestingly, the enhancing properties of TRIM11 (human and mouse) and mouse TRIM30 proteins were specific for N-MLV and not observed for B-MLV. The ratio of fold inhibition for N versus B-MLV as well as the scatter plot analysis illustrate this finding (Figure 1E and 1G). Thus, the inhibitory effects of TRIM1 and 5 as well as the enhancing effects of TRIM11 (human and mouse) and TRIM30 (mouse) strongly correlate with N-tropism.

To validate critical results gained in our transient expression screen, we downregulated endogenously expressed human TRIM proteins 11, 25, 31 and 62 in HeLa cells using RNA interference (RNAi) (Figure 1H). Consistent with the inhibitory effects of TRIM 11 and 31 on HIV entry, downregulation of both proteins using siRNA facilitated HIV entry (2–3-fold). While modest, these enhancements in viral entry suggest that TRIM11 and 31 contribute to the restriction of HIV in HeLa cells. Likewise, the enhancing effect of TRIM11 expression on N-MLV entry led to a corresponding inhibition following gene downregulation of the endogenous protein (3-fold). In contrast, TRIM25 and 62 exhibited inhibitory effects against MLV viruses when overexpressed or silenced using RNAi. Thus, the transient expression screen for TRIM proteins identified human TRIM11 and TRIM31 as factors modulating retroviral entry.

### A Role for TRIM 11 in the Reduction of TRIM 5 Protein Levels

**Figure 2:**
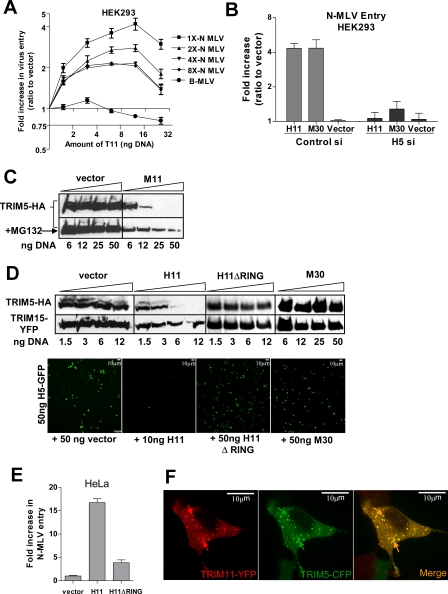
TRIM11 Reduces the Protein Levels of TRIM5 (A) Effects of increasing amounts of transfected plasmid encoding human TRIM11 on entry of B-MLV and increasing amounts of N-MLV. (B) The effects of transient expression of TRIM11 (10 ng DNA transfected), TRIM30 (50 ng DNA transfected), and empty vector (Vector) on N-MLV entry were tested in HEK293 cells treated with either control (control si) or TRIM5 specific siRNA (H5 si). (C) Western blot (anti-HA serum) of HEK293 cells 48 h after transfection with plasmids encoding HA-tagged TRIM5 and either increasing amounts (ng DNA) of empty vector (pcDNA) or a plasmid expressing mouse TRIM11 (M11). Cells were treated with the proteasome inhibitor MG132 (10 μM) for 6 h prior to analysis as indicated. (D) An experiment as in (C) for cells expressing HA-tagged TRIM5 or TRIM15-YFP together with human TRIM11 (H11), its mutant lacking the RING domain (ΔRING) and mouse TRIM30 (M30). The lower panel shows the parallel TRIM5 GFP-levels in cells. (E) Fold increase in N-MLV entry in the HeLa cells expressing either control plasmid, wild-type human TRIM11, or its RING domain mutant (ΔRING). (F) TRIM11-YFP (5 ng, red) and TRIM5-CFP (50 ng, green) were transfected into HEK293 and the localization of both proteins monitored by confocal microscopy 24 h post-transfection.

The specific enhancement of N-MLV, but not B-MLV entry upon expression of TRIM11 proteins as well as mouse TRIM30 was unanticipated (Figure 1C–1E). These enhancing properties of TRIM11 were observed over a wide range of expression levels (Figure 2A). Silencing of endogenous TRIM11 by siRNA in HeLa or HEK293 cells led to an increase in restriction of N-MLV, but not B or NB-MLV (Figure 1H and data not shown). Together, these data suggest that TRIM11 regulates the Ref1 restriction in human cells. HEK293 cells endogenously express low levels of human TRIM5 that restricts N-MLV entry [10–12].

To test if the effects of TRIM11 and TRIM30 on N-MLV entry are due to interference with the Ref1 restriction, we silenced TRIM5 in HEK293 cells by RNAi. Indeed, the enhancing effects of TRIM11 and TRIM30 on N-MLV entry were dependent on the presence of TRIM5 and lost in response to TRIM5 silencing (Figure 2B).

Potentially, these proteins affect TRIM5 protein levels. To test this hypothesis for TRIM11, a plasmid encoding HA or GFP-tagged TRIM5 was co-transfected together with either empty vector or increasing amounts of a plasmid encoding mouse and or human TRIM11. TRIM5 protein levels were then examined by western blot and fluorescence microscopy (Figure 2C and 2D). Strikingly, expression of low amounts of TRIM11 led to the disappearance of TRIM5, an effect that could be delayed by treating cells with the proteasome inhibitor MG132 (Figure 2C and 2D). The reduction of TRIM5 levels as determined by western blotting corresponded with a loss of cytoplasmic bodies (Figure 2D, lower panel). The protein levels of another TRIM protein, human TRIM15, were largely unaffected by the expression of human TRIM11 (Figure 2D). Because both proteins are expressed from the same promoter, the observed reduction in TRIM5 levels is likely not explained by effects of TRIM11 on transcription. Deleting the RING domain of TRIM11 did not affect TRIM5 protein levels, indicating a functional dependence on E3 ligase activity (Figure 2D). Correspondingly, entry of N-MLV in HeLa cells was enhanced 16-fold by the expression of wild-type TRIM11, whereas TRIM11 lacking the RING domain exhibited reduced activity (Figure 2E). Together these results suggest that TRIM11 regulates the turnover of TRIM5 thereby regulating the level of Ref1 restriction in mammalian cells. The observed co-localization of TRIM11 and TRIM5 proteins to cytoplasmic bodies is consistent with such a model (Figure 2F).

In contrast to TRIM11 proteins, expression of mouse TRIM30 did not affect the protein levels of TRIM5 (Figure 2D). How mouse TRIM30 interferes with the Ref1 restriction remains to be determined. Interestingly, TRIM30 is the closest homologue of human TRIM5 in the mouse genome, but carries a deletion in the variable region 1 within the B30.2 domain that is critical for interaction with capsid (Figure S1D) [30–32]. Mouse TRIM30 may function as a dominant-negative protein not unlike TRIM5 proteins lacking the B30.2 domain [8,31].

### Antiviral Activities of TRIM Proteins Affecting Late Stages of Retroviral Replication

**Figure 3:**
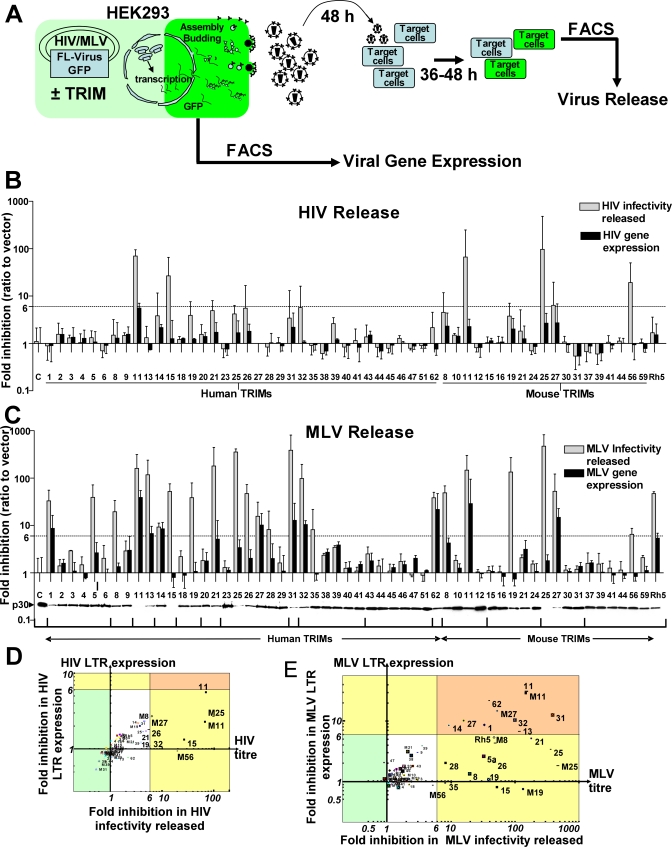
TRIM Proteins Interfering with Virus Production and Release (A) Experimental design: HEK293 cells transfected with viral plasmids encoding full-length HIV or MLV and carrying a GFP-reporter gene in the presence or absence of plasmids encoding individual TRIM proteins. 48 h after transfection, producer cells and culture supernatants were harvested. Producer cells were subjected to FACS analysis to measure viral gene expression. The infectivity of culture supernatants was determined by infecting target cells and measuring GFP-positive cells 36 h after initiation of infection using FACS analysis. (B, C) Effects of TRIM protein expression in HEK293 cells on the production and release of HIV (B) and MLV (C). Graphs represent the fold inhibition (log10 scale) in released viral infectivity (grey bars) and in viral gene expression (black bars). All experiments were performed at least in triplicates and the bars represent standard errors from the mean values. The dotted line represents the statistically determined cut-off value of 6. MLV Gag released into culture supernatant was analysed by western blot using antibodies against p30 MLV capsid and presented beneath the graph in (C). Partition lines indicate individual gels. (D, E) Scatter plots of data shown in panels (B) and (C) depicting fold inhibitions in released virus infectivity plotted against viral gene expression from the LTR upon TRIM expression for HIV (D) and MLV (E).

After studying the role of TRIM proteins during early events in viral replication, we next investigated if they exhibited antiviral effects at late stages of viral replication (Figure 3A). To determine effects specific for HIV release, we bypassed entry by directly transfecting plasmids that encoded for TRIM proteins along with a HIV variant HXB2ΔEnv-GFP lacking Env and expressing cytoplasmic GFP. Infectious virions were generated by co-expression of VSVG. After 48 h, the culture supernatants were harvested and the level of GFP expression in producer cells was determined by flow cytometry. The viral infectivity in the harvested culture supernatant was determined by infecting susceptible target cells and measuring GFP-positive cells after an additional 36 h. In addition, the Gag protein released into the supernatant was determined by western blot using antibodies to the HIV capsid protein p24. The results of such an experiment for HIV release is shown in Figure 3B in fold inhibition. HIV capsid released into the supernatant is presented in Figure S2A. Our analysis identified the human TRIM proteins 15, 26, 32, the mouse proteins 11, 25, 27, and 56 as factors that specifically affected HIV release from cells, but not viral gene expression (Figure 3B). A number of TRIM proteins (human TRIM19, 21, 25 and mouse TRIM8) were close to the statistically determined cut-off value of 6. At the transfection level (50ng) used in the assembly assay, human TRIM11 affected both viral gene expression and virus release. A scatter plot analysis depicting the effects of TRIM protein expression on virus release over effects on LTR expression summarizes these results (Figure 3D).

We then performed an identical experiment for NB-tropic MLV (Figure 3C) using a plasmid encoding for the Friend57 MLV genome carrying a GFP insertion into the Env protein [33,34]. MLV capsid (p30) released into the supernatant is presented below the graph (Figure 3C). Our analysis revealed a striking sensitivity of MLV to the expression of TRIM proteins, in particular human TRIM proteins. Overall, 21 TRIM proteins and rhesus TRIM5 inhibited MLV release at least 10-fold, 9 of which inhibited MLV release by more than 100-fold (Figure 3C). A dose-response experiment revealed that transfecting small amounts of plasmids encoding for human TRIM15, 25, 31, 62 and mouse 11 and 25 resulted in potent antiviral activity (Figure S2B). In contrast, higher DNA amounts were required for the other TRIM proteins, particularly TRIM5. TRIM22, while removed from the screen due to varying results, did exhibit antiviral activity in a dose-dependent manner.

Generally, we observed two distinct phenotypic groups for MLV restriction. The first group, including the TRIM proteins 8, 15, 19, 25, 26, 28 and 35 specifically interfered with the release of infectious MLV into the culture supernatant without major effects on viral gene expression (Figure 3C). For the second group, consisting of human TRIM proteins 1, 11, 13, 14, 21, 27, 31, 32, 62, the mouse proteins 8, 11, 27 and rhesus 5, the inhibitory effects on viral gene expression were close to the cut-off of 6 or higher implying that a suppression of viral gene expression contributed to the observed reduction in the release of infectivity. A scatter plot depicting effects of TRIM proteins on infectivity versus gene expression summarizes these results (Figure 3E).

For a number of TRIM proteins, the reduction in infectivity did not correspond with a proportionate reduction in p30 release. Western blot analysis of released virus for Env and Gag revealed that human TRIM13, 21 and mouse TRIM19 preferentially affected Env incorporation (Figure S2C). A reported role for TRIM13 in protein degradation at the endoplasmic reticulum is consistent with such a phenotype [35].

Finally, to measure ALV release, we studied the effects of TRIM expression in chicken fibroblasts because the release of this virus is restricted in mammalian cells [36]. Among the few potent antiviral proteins were human and mouse TRIM25, human 32 and mouse TRIM11 (Figure S2D). Human TRIM11 was not tested for ALV.

### Gene Silencing of Human TRIM Proteins 25, 31, and 62 Affects HIV and MLV Release

**Figure 4:**
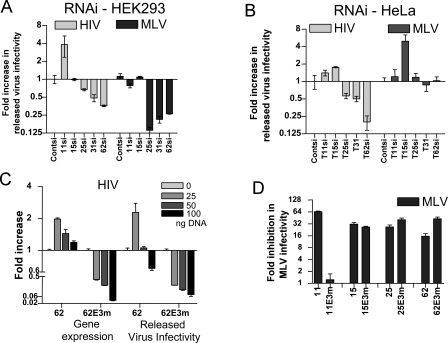
Inhibitory and Enhancing Roles of Individual TRIM Proteins on Viral Release (A, B) Fold increase in HIV and MLV infectivity released from HEK293 (A) and HeLa (B) cells treated with control siRNA (contsi) or siRNA targeting endogenously expressed human TRIM proteins 11, 15, 25, 31, and 62. (C) Fold increase in HIV gene expression and released infectivity in HEK293 cells transfected with increasing amounts of plasmid (0–100 ng) expressing TRIM62 or its E3 mutant (62E3m). (D) Infectivity of MLV released from HEK293 cells is shown as fold inhibition in presence of plasmids expressing TRIM11, 15, 25, 62, or their E3 mutants (E3m).

The inhibitory effects observed above could be the result of overexpression of these proteins in HEK293 cells. To determine their role in the viral life cycle, we targeted endogenously expressed TRIM proteins for downregulation using RNAi. We concentrated on TRIM 11, 15, 25, 31 and 62 because they were very effective at low transfection levels and were endogenously expressed in HEK293 and HeLa cells (Figure S3A and S3B). Downregulation of TRIM proteins 25, 31 and 62 inhibited HIV and MLV release in HEK293 cells, suggesting that these proteins play a role in efficient virus release (Figure 4A and 4B). Notably, silencing of TRIM62 also strongly interfered (7-fold) with HIV release in HeLa cells (Figure 4B). Correspondingly, expression of low amounts of TRIM62 enhanced HIV gene expression and release (Figure 4C).

To gain further insights into the antiviral activities of these proteins we tested mutant proteins impaired in their E3 ligase activity. Interestingly, the E3 mutant of TRIM62 inhibited HIV and MLV release more potently than the wild-type protein, likely by exhibiting a pronounced dominant-negative effect (Figure 4C and 4D). Together, these data suggest that TRIM 25, 31 and 62 play an important role in virus release.

### TRIM11 and 15 Contribute to the Innate Restriction of Retroviruses in Human Cells

In contrast to TRIM25, 31 and 62, downregulation of endogenous TRIM11 enhanced HIV release ∼4-fold in HEK293 and moderately (2×fold) in HeLa cells (Figure 4A and 4B). MLV release was enhanced 5-fold in response to silencing of TRIM15 in HeLa cells (Figure 4B). The enhancement of virus release observed in response to gene silencing is consistent with the hypothesis that both TRIM proteins contribute to the endogenous restriction of HIV and MLV in mammalian cells.

We next tested the contribution of E3 ligase function to the antiviral activity of both proteins. Interestingly, the antiviral activity of TRIM11 was critically dependent on a functional E3 ligase domain (Figure 4D) implying the involvement of the ubiquitin-dependent degradative pathway. In contrast, the E3 mutant of TRIM15 largely retained its inhibitory activity indicating that it interferes with viral replication via a different mechanism (Figure 4D).

### The Antiviral Activity of TRIM15 Depends on the Ability of its B-box to Interact with the MLV Gag Precursor Protein

**Figure 5:**
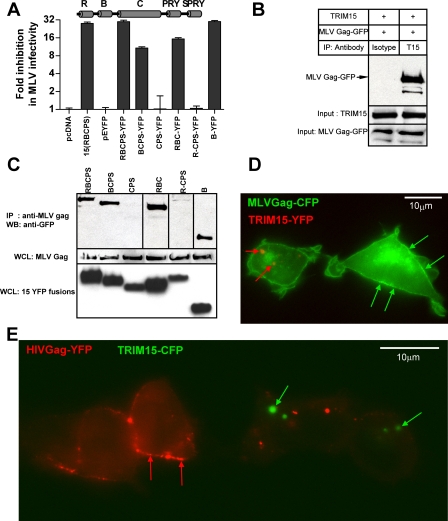
The Antiviral Activity of TRIM15 Resides in Its B-box (A) Fold inhibition in MLV released infectivity in presence of indicated TRIM15-YFP derivatives. The vectors pcDNA or pEYFP were used as controls. Top schematic displays the TRIM15 domain structure. (B) Western blot using GFP antibodies to detect MLV Gag-GFP that co-immunoprecipitated with antibodies against TRIM15. (C) Western blot using antibodies to GFP to identify TRIM15-YFP derivatives that co-immunoprecipitated with MLV Gag. (D) MLV Gag-CFP (green), full length MLV and TRIM15-YFP (red) were transfected into HEK293 cells and monitored 24 h later. Green arrows indicate the accumulation of Gag at the plasma membrane. Red arrows point to cytoplasmic TRIM15 bodies. (E) An experiment as described in (D) was performed for HIV Gag-YFP (red) and TRIM15-CFP (green).

To understand how TRIM15 can interfere with viral release in an E3 ligase independent manner, we performed a domain analysis for human TRIM15. TRIM15 YFP fusion protein was as active as its untagged version. Hence this analysis was performed using YFP fusion proteins (Figure 5A). Interestingly, TRIM15 lacking the B-box, but not RING or SPRY domains, lost all of its antiviral activity (Figure 5A). In fact, the B-box alone exhibited antiviral activity.

TRIM5 specifically interferes with retroviral entry by binding to incoming mature capsids [13–15] and it had recently been suggested that rhesus TRIM5 can also bind and degrade immature capsids interfering with virus production [37]. To test if TRIM15 can bind to the immature Gag precursor protein of MLV, we performed co-immunoprecipitations. Interestingly, antibodies against TRIM15 specifically co-immunoprecipitated MLV Gag and vice versa (Figure 5B and 5C). Importantly, TRIM15 fragments containing the B-box interacted with MLV Gag, while TRIM15 mutants lacking the B-box did not. In fact, the B-box alone was capable of interacting with the MLV Gag precursor protein. Thus, TRIM15 interferes with MLV release by directly or indirectly binding the MLV Gag precursor protein via its B-box.

To understand how TRIM15 binding to Gag alters the cellular fate of retroviral capsids, we transfected plasmids encoding for TRIM15-YFP together with MLV Gag-CFP and replication competent MLV into HEK293. Visualization using fluorescence microscopy revealed on average a reduction of Gag fluorescence at the plasma membrane in cells containing cytoplasmic TRIM15 bodies (Figure 5D). When TRIM15-CFP was expressed together with HIVGag-YFP, a similar phenotype was observed. Less HIV Gag reached the plasma membrane, but rather accumulated intracellularly (Figure 5E).

## Discussion

Using a transient expression screen in HEK293 cells we have performed the first near comprehensive screen for antiviral activities of members of the TRIM family of proteins. Our screen identifies ∼20 TRIM proteins with antiviral activity demonstrating that multiple TRIM proteins can exhibit antiviral activities. Because many TRIM proteins are upregulated in response to interferons [2], a potential role of these proteins in the establishment of an antiviral state should be investigated.

**Figure 6:**
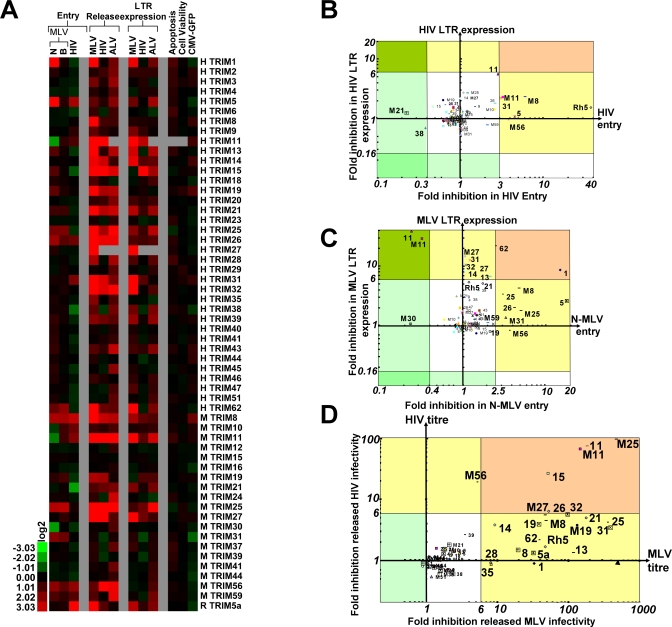
Anti-retroviral Activities of TRIM Proteins Identified in This Study (A) A Java TREEVIEW graphic display of all TRIM proteins activities against early and late stages of the retroviral life cycle. Input fold inhibition values were log2 transformed and shown as red/green color-coding for inhibitory and enhancing activities, respectively. Grey stands for experiments not carried out. (B–D) Scatter plot analyses were performed as indicated.

The specific effects of TRIM proteins on the replication cycle of each retrovirus are summarized in Figure 6A using red/green color-coding for inhibitory and enhancing activities, respectively. This presentation readily displays the specificity of TRIM1, 5, 11 and 30 for N-MLV entry. This analysis also allows a comparison of the entry results with the viral gene expression data obtained in the screen for virus release (Figure 6A–6C). For example, the inhibitory activities of TRIM1 and 62 on N-MLV entry likely include effects on viral gene expression (Figure 6A and 6C).

This analysis also reveals that an overexpression of human and mouse TRIM11 proteins affects MLV gene expression (Figure 6A and 6C). Despite these inhibitory effects, TRIM11 expression specifically enhanced N-MLV, but not B-MLV entry. Under these conditions, the protein levels of transiently expressed TRIM5 were reduced. TRIM11 may contribute to the turnover of endogenous TRIM5, because silencing of endogenous TRIM11 enhanced the Ref1 restriction. These two observed activities of TRIM11, degradation of cytoplasmic proteins as well as the regulation of transcription, are similar to previous reports for a role of TRIM11 in the turnover of humanin and ARC105 [38,39]. Clearly, several cellular targets exist for TRIM11. Its potential role in the turn over of TRIM5, a protein that potently restricts retrovirus entry, could be of therapeutic importance.

Strikingly, most of the antiviral TRIM proteins exhibited strong inhibitory effects against late stages of the viral life cycle (Figure 6A). MLV was highly sensitive to the expression of TRIM proteins, particularly of human origin. Of the 14 TRIM proteins specifically interfering with MLV release, only two were of mouse origin (Figure 6D). A similar cross species effect was observed for HIV. 21% of all mouse, but only 11% of all human TRIM proteins interfered with HIV release. Cross species effects are consistent with the hypothesis that TRIM proteins contribute to the innate control of retroviruses and that over time, viruses can adapt to inhibitory effects.

In our further analysis we concentrated on TRIM proteins that were highly effective even at very low transfection levels. Gene silencing of TRIM 25, 31 and 62 inhibited virus release suggesting that these E3 ligases play a role in cellular pathways critical for virus release. They were identified in our expression screen likely because overexpression of the wild-type protein exhibited a dominant-negative effect.

In contrast, downregulation of TRIM11 and TRIM15 enhanced virus release suggesting that these proteins contribute to the restriction of MLV and HIV even in highly susceptible HEK293 and HeLa cells. A detailed understanding of host restriction may lead to antiviral therapies aimed at strengthening the innate immunity to retroviruses at the cellular level. The interaction of TRIM15 with retroviral Gag suggests that TRIM proteins, apart from entry, can recognize retroviral Gag proteins during assembly and budding and thereby inhibit viral release. A preferential targeting of late stages of the retroviral life cycle may be more consistent with a role for TRIM proteins in the establishment of an antiviral state.

## Materials and Methods

### Cell lines and plasmids.

HEK293, HeLa, DFJ8 and DF-1 were described previously [40]. TZM-bl cells were a gift from Vineet KewalRamani (NCI Frederick, MD). TRIM constructs presented in Table 1 were confirmed by sequencing to be authentic and in the correct reading frame. The reference for human TRIM22 is NM_006074. YFP fusion proteins of human TRIM 11, 15, 25, 31 and 62 were generated by insertion of PCR amplified genes into the EcoRI/XhoI sites of pEYFP-N1 (Clontech, Palo Alto, CA). E3 mutants were created by substituting two active site cysteines to alanine using site-directed mutagenesis (QuikChange, Stratagene, La Jolla, CA). TRIM15-YFP mutants were generated by PCR; BCPS, CPS, RBC, R-CPS-YFP fusions lacked the amino acids 1–64, 1–119, 346–465 and 81–119, respectively. The B-box-YFP corresponds to amino acids 64–129.

### Generation of viruses.

N, B and NB-tropic MLV were prepared by transfecting 4 μg of a plasmid encoding a viral RNA (pLZRS-GFP) [40], 4 μg plasmid encoding the envelope glycoprotein of subgroup A of ALV (EnvA) [41] and 4 μg of plasmids expressing either N-, B-tropic (pCIG3-N or B, gifts from Greg Towers and Jonathan Stoye, University College London, UK) [42] or NB GagPol (pMDGag-Pol) [41] into a 10 cm plate of HEK293 cells using FuGene 6 (Roche, Indianapolis, IN, USA) and serum-free OPTIMEM media (Invitrogen Corporation, California). HIV-1 reporter viruses were generated by transfecting 4 μg of HXB2Env^−^-GFP, a HXB2 derivative (lacking envelope and encoding GFP instead of nef gene; gift from Heinrich Gottlinger, Worcester, UMass, MA), and 4 μg plasmid encoding ALV EnvA [41]. ALV reporter viruses were from supernatants of DF-1 cells chronically infected with RCASBP(A)-GFP [41]. For siRNA experiments, viruses were generated carrying the Vesicular stomatitis virus G (VSVG) envelope protein instead of ALV-A Env. The culture medium was harvested 48 h after transfection, filtered through 0.45 μm, aliquoted and stored at −80 C. To determine the titer, serial dilutions of virus stocks were titrated onto DFJ8 cells in the presence of 5 μg/ml of polybrene followed by flow cytometry of GFP- positive cells (FACS, Becton Dickinson) 36–48 h later.

### Virus entry assays.

HEK293 cells were co-transfected in 24 wells with 50 ng each of plasmids encoding the TRIM protein and 25 ng of ALV receptor Tva950. 30 h after transfection, cells were seeded into 48-well plates at a density of 1.5 × 10^4^ target cells/well. After an additional 6 h, cells were challenged with N, B-MLV, HIV or ALV carrying a GFP reporter genome. GFP-positive cells were quantified by flow cytometry after 36 h post infection. To perform the screen initially with a dynamic range that allows reliable detection of both inhibiting and enhancing effects, an amount of virus was used that resulted in infection levels of 5%. Strong inhibitory or enhancing TRIM proteins were characterized in a second round with adjusted infection levels. All experiments were at least performed four times on separate days. These data sets were combined for the final analysis shown in Figure 1. Fold inhibitions in virus entry represent the ratio of percent GFP-positive cells of cells transfected with empty control vector versus those expressing TRIM proteins. The maximum variability between control samples in the absence of TRIM proteins was ∼1.5-fold. The cut-off value of 2.5 applied in Figure 1 was the derived from 1.5 plus two times the standard deviations (confidence value of 95%) of 0.5.

### Virus release and gene expression assays.

2.5 × 10^5^ HEK293 cells (producer cells) in 24-wells were transfected with 50 ng TRIM expressing construct with either 150 ng each of the HXB2Env^−^-GFP and 50 ng plasmid expressing VSVG. For MLV release assays, 200 ng of plasmid MLVEnv-GFP encoding full length Friend 57 MLV genome with a GFP insertion into the envelope protein [33,34] was co-transfected with TRIM expressing construct as above. ALV release assays were conducted by transfecting 3 × 10^4^ DF-1 cells in 24-well plate with 50 ng TRIM expressing plasmids, 200 ng of ALV vector lacking the envelope protein (plasmid DASBP-GFP, a gift from Stephen Hughes, NCI Frederick, MD) and 50 ng of plasmid expressing ecotropic Friend MLV envelope protein (pcDNA3-FrEnv) [33]. 48 h after transfection, the released virus infectivity was measured by applying two dilutions of the culture supernatants differing by 10-fold onto target cells (DFJ8 for MLV and ALV; HEK293 or TZMbl cells for HIV) in presence of 5 μg/ml of polybrene. GFP-positive cells were enumerated after additional 36–48 h as above. For measuring viral gene expression, the mean fluorescence intensity (MFI) of GFP in the transfected producer cells (48 h after transfection) was estimated using FACS (Becton Dickinson). Fold inhibition in viral gene expression was calculated using the ratio of GFP MFI in cells transfected with vector to those expressing TRIM. The maximum variability between control samples in the absence of TRIM proteins was ∼2-fold. The cut-off value of 6 applied in Figure 3 was the derived from 2 plus two times the standard deviations (confidence value of 95%) of 2.

To measure the release of Gag (p30 for MLV and p24 for HIV) a parallel experiment as described above was conducted in triplicates. 48 h after transfection the culture supernatants from triplicate wells were combined and viruses sedimented at 12,000 × g in a microcentrifuge for 2 h. The resulting 12,000 g pellet was solubilized in SDS gel loading buffer and resolved on SDS-10% PAGE followed by western blot using antibodies to MLV capsid and Env (p30 & gp70; Quality Biotech, Camden, NJ) or HIV capsid (p24; obtained through the AIDS Research and Reference Reagent Program, NIH from Dr. Michael H. Malim.)

### Cell viability, apoptosis, and gene expression assays.

Cell viability and Caspase 3/7 activity was measured sequentially for the same samples 48 h after transfection of 50 ng TRIM-expressing construct in HEK 293 cells using CellTiter-Blue™ Cell Viability and Caspase-Glo 3/7 Assay System (Promega Corp.) according to manufacturer's recommendations. To measure the effect on cellular gene expression due to transient expression of TRIM proteins, 10 ng of plasmid expressing GFP under a CMV promoter was co-transfected with 50 ng of TRIM encoding plasmids in HEK293 cells. 48 h later the MFI of GFP and fold inhibitions were calculated as above.

### Viral entry and release assays in RNAi treated cells.

2 × 10^5^ HeLa or HEK293 cells in 48 well plates were transfected with 80 nM TRIM-specific siRNA smartpool (Dharmacon Inc) or control siRNA (Dharmacon Inc, #D-001210–01) using Lipofectamine 2000 (Invitrogen, CA). After 4 h the medium was changed and after additional 20 h the cells were split 1:4 into 4 wells of a 48 well plate. For entry assays, 36 h post-transfection, the cells were challenged as above with VSV-G pseudotyped reporter N-, NB-MLV and HIV at two concentrations of virus differing by 2-fold. After additional 24 h the cells were harvested, fixed and analyzed by FACS. For virus release assays, 30 h after first siRNA transfection, cells were transfected again with 80 nM TRIM-specific siRNA smartpool or control siRNA together with either 200 ng plasmid encoding full length Friend MLV genome carrying a GFP insertion into the envelope protein or 100ng of full length replication competent HIV-1 plasmid pNL4–3 (AIDS Research and Reference Reagent Program). After an additional 48 h, the culture supernatants were harvested and applied onto DFJ8 for MLV and TZMbl cells for HIV titration. For MLV, DFJ8 cells were harvested 48 h after infection and analyzed by FACS as before. For HIV, 48 h after infection, TZMBL cells were lysed and luciferase activity measured using a Luminometer (Turner Biosystems). Fold increase in entry or release was calculated using a ratio of the percent GFP positive cells or luciferase activity (measured as relative light units) from experimental samples transfected with TRIM specific siRNA and those transfected with control siRNA.

### TRIM specific siRNA used in the study.

Silencing of endogenous human TRIM proteins was carried out using ON-TARGET*plus* siRNA smart pools (a mix of 4 siRNAs) from Dharmacon, Inc. pre-designed to reduce off-target effects by up to 90%. We routinely obtained 70 to 90% knock down of specific TRIM proteins as assessed by monitoring the levels of transiently expressed TRIM-GFP 36 h post-transfection. The sense sequences of siRNAs targeting (1) TRIM11 were #1: AG GCGAAGCUGGAGAAGUCUU, #2: GAGCUGAUC CUGUCUGAAGUU, #3: UCACUGCUA UUCAUCUUUCUU, #4: GGACAGCCCAGAGCGCU UUUU; (2) TRIM15 were #1: GGGAGAAACUUACUGCGAGUU, #2: GCGAGAACGAUGCCG AGUUUU, #3: CCCUGAAGGUGGUCCAUGAUU, #4: GCAGAACCACAGACGGCUUUU; (3) TRIM25 were #1: CGGAACA GUUAGUGGAUUUUU, #2: CAACAAGAAUACACGGAA AUU, #3: GCGGAUGACUGCAAACAGAUU, #4: GGGAUGAGUUCGAGUUUCUUU; (4) TRIM31 were #1: GGAGAAGAAUU UCCUGCUAUU, #2: GGAAGAACGCAAUCAGGUU UU, #3: AAUUUGAACUCCUGCAUCAUU, #4: CCACAAAUCCCAUAAUGUCUU; (5) TRIM62 were #1: CUACAAUGCUGAUGACAUGUU, #2: GCGAGAAGUUCCCUGGCAAUU, #3: AGACCAACCUCACAUAUGAUU, #4: GACCAAGUCUUCCACCAAGUU. For human TRIM5, the sequences of the siRNA smart pool from Dharmacon were as previously reported [8]. TRIM5 silencing using this smart pool resulted in a ∼4-fold and ∼50-fold enhancement of N-MLV entry in HEK293 and HeLa cells, respectively.

### Data analysis.

For easy interpretation of the screen we used scatter plots generated using Excel. Fold inhibitions for the two parameters compared were plotted against each other in log scale. Java TREEVIEW was used to represent data in color codes [43]. The input fold inhibition values obtained as described in previous sections were log2 transformed to obtain positive (inhibition, shades of red) and negative (enhancement, shades of green) values prior to data analysis using TREEVIEW.

### Co-immunoprecipitation analysis.

HEK293 cells were transfected as above with 50 ng of plasmids encoding TRIM15 derivatives with C-terminal YFP fusions or untagged TRIM15 and 200 ng of plasmid encoding full length Friend MLV genome carrying a GFP insertion into the envelope protein or 100 ng of MLV Gag-GFP. 48 h post-transfection, the cells were lysed using triple detergent lysis buffer (TDLB, 100 mM Tris [pH 8.0], 1% Triton-X-100, 0.5% sodium deoxycholate, 0.2% sodium dodecyl sulphate, 150 mM NaCl). The nuclei and undissolved cellular components were removed by centrifugation at 12,000 × g for 30 minutes in a microcentrifuge. The clarified 12,000 × g supernatant was used for immunoprecipitation using protein-G beads prebound with antibodies to MLV capsid (Quality Biotech, Camden, NJ) or TRIM15 (Abcam, Boston, MA) raised in goat or isotype specific antibodies. The immunoprecipitates were washed three times with TDLB and analyzed using SDS-10%-PAGE followed by western blot using antibodies to GFP.

### Imaging.

The generation of fluorescently labeled MLV and HIV virions using Gag-GFP proteins was previously described [33]. To visualize MLV Gag and TRIM15 in HEK293 cells, plasmids encoding for MLV Gag-CFP (50 ng), replication competent MoMLV (200 ng) and TRIM15-YFP (10 ng) were co-transfected. To perform a similar experiment for HIV, 50 ng HIV Gag-YFP was co-transfected with 10 ng TRIM15-CFP. 24 h later, cells were fixed and the CFP and YFP channels monitored using the 60x oil objective (NA 1.4) of a Nikon TE2000 inverted wide-field microscope. To monitor TRIM11 and TRIM5, HEK293 cells were transfected with 5 ng TRIM11-YFP together with 50 ng TRIM5-CFP and cells imaged 24 h post-transfection using the 60× oil Nikon objective (NA 1.4) and an Improvision spinning disc confocal microscope.

### Reverse-transcription PCR for detection of endogenous expressed TRIMs.

Total RNA was extracted from HEK293 and HeLa cells using PrepEase RNA extraction kit (USB), which has an on-column DNAse treatment step. The RNA was reverse transcribed with anchored oligodT using Reverse-iT first strand synthesis kit (ABgene). The cDNA was then used to check presence TRIM-specific sequences by PCR using appropriate primer pairs. Control reactions which used plain RNA for PCR amplification did not yield any products (data not shown). Specific primer pairs used for amplifications were obtained from Primer Bank database and sequences can be found at http://pga.mgh.harvard.edu/primerbank/index.html.

## Supporting Information

Figure S1 Effects of TRIM Protein Expression on Cell Viability and CMV Promoter Activity (A) The effect of TRIM protein expression in HEK293 cells using 50 ng of TRIM-expressing constructs in a 24 well on the induction of apoptosis was monitored 48 h post-transfection using a caspase 3/7 activity assay.
(B) An assay as in (A) was performed to measure the effects of TRIM protein expression on cell viability using the ability of cells to convert resazurin into resorufin.
(C) The effects of TRIM protein expression in HEK293 cells as in (A) on the activity of the CMV promoter were tested by measuring GFP fluorescence of the plasmid pEGFP-N1. For TRIM11 proteins, the transfection of 10 ng reduced pleiotropic effects on transcription.
(D) Sequence comparison of the B30.2 domain of mouse TRIM30 in comparison to its closest homologues rat TRIM30 (68% identity), the TRIM5 like proteins from cow 505265 (48%) and pig 733579 (47%), human TRIM5 (47%), and rhesus TRIM5 (45%).
(1.3 MB TIF)

Figure S2 TRIM Proteins Interfere with Retroviral Release (A) HIV p24 capsid released into the culture supernatant from HEK293 cells expressing indicated TRIM proteins. Effects of TRIM protein expression on HIV release and viral gene expression are as in Figure 3B.
(B) TRIM proteins interfere with MLV release in a dose dependent fashion. An experiment as in Figure 3C was performed with increasing amounts of transfected plasmids encoding TRIM proteins.
(C) MLV p30 capsid and gp70 Env released into the culture supernatant from HEK293 cells expressing indicated TRIM proteins.
(D) An experiment as in Figure 3 was performed to determine TRIM proteins that interfere with ALV release in DF-1 cells.
(3.7 MB TIF)

Figure S3 Endogenous Expression and Silencing of TRIM Proteins Expressed in HEK293 and HeLa Cells (A) Reverse transcriptase PCR for the presence of indicated TRIM mRNA in HEK293 and HeLa cells.
(B) Western blot using GFP antibodies for indicated TRIM-YFP fusion proteins expressed in the presence of control (con) or specific siRNA (sp). Protein loads were monitored using antibodies against actin.
(1.9 MB TIF)

## Abbreviations

ALV: avian leukemia virus
GFP: green fluorescent protein
HIV: human immunodeficiency virus
MLV: murine leukemia virus
TRIM: TRIpartite interaction Motif

## References

[ppat-0040016-b001] Goff SP (2007). Host factors exploited by retroviruses. Nat Rev Microbiol.

[ppat-0040016-b002] Nisole S, Stoye JP, Saib A (2005). TRIM family proteins: retroviral restriction and antiviral defence. Nat Rev Microbiol.

[ppat-0040016-b003] Towers GJ, Goff SP (2003). Post-entry restriction of retroviral infections. AIDS Rev.

[ppat-0040016-b004] Bieniasz PD (2004). Intrinsic immunity: a front-line defense against viral attack. Nat Immunol.

[ppat-0040016-b005] Sokolskaja E, Luban J (2006). Cyclophilin, TRIM5, and innate immunity to HIV-1. Curr Opin Microbiol.

[ppat-0040016-b006] Best S, Le Tissier P, Towers G, Stoye JP (1996). Positional cloning of the mouse retrovirus restriction gene Fv1. Nature.

[ppat-0040016-b007] Pincus T, Hartley JW, Rowe WP (1975). A major genetic locus affecting resistance to infection with murine leukemia viruses. IV. Dose-response relationships in Fv-1-sensitive and resistant cell cultures. Virology.

[ppat-0040016-b008] Stremlau M, Owens CM, Perron MJ, Kiessling M, Autissier P (2004). The cytoplasmic body component TRIM5alpha restricts HIV-1 infection in Old World monkeys. Nature.

[ppat-0040016-b009] Sayah DM, Sokolskaja E, Berthoux L, Luban J (2004). Cyclophilin A retrotransposition into TRIM5 explains owl monkey resistance to HIV-1. Nature.

[ppat-0040016-b010] Hatziioannou T, Perez-Caballero D, Yang A, Cowan S, Bieniasz PD (2004). Retrovirus resistance factors Ref1 and Lv1 are species-specific variants of TRIM5alpha. Proc Natl Acad Sci U S A.

[ppat-0040016-b011] Keckesova Z, Ylinen LM, Towers GJ (2004). The human and African green monkey TRIM5alpha genes encode Ref1 and Lv1 retroviral restriction factor activities. Proc Natl Acad Sci U S A.

[ppat-0040016-b012] Yap MW, Nisole S, Lynch C, Stoye JP (2004). Trim5alpha protein restricts both HIV-1 and murine leukemia virus. Proc Natl Acad Sci U S A.

[ppat-0040016-b013] Perron MJ, Stremlau M, Lee M, Javanbakht H, Song B (2007). The human TRIM5alpha restriction factor mediates accelerated uncoating of the N-tropic murine leukemia virus capsid. J Virol.

[ppat-0040016-b014] Stremlau M, Perron M, Lee M, Li Y, Song B (2006). Specific recognition and accelerated uncoating of retroviral capsids by the TRIM5alpha restriction factor. Proc Natl Acad Sci U S A.

[ppat-0040016-b015] Sebastian S, Luban J (2005). TRIM5alpha selectively binds a restriction-sensitive retroviral capsid. Retrovirology.

[ppat-0040016-b016] Reymond A, Meroni G, Fantozzi A, Merla G, Cairo S (2001). The tripartite motif family identifies cell compartments. EMBO J.

[ppat-0040016-b017] Meroni G, Diez-Roux G (2005). TRIM/RBCC, a novel class of ‘single protein RING finger' E3 ubiquitin ligases. Bioessays.

[ppat-0040016-b018] Everett RD, Chelbi-Alix MK (2007). PML and PML nuclear bodies: Implications in antiviral defence. Biochimie.

[ppat-0040016-b019] Campbell EM, Dodding MP, Yap MW, Wu X, Gallois-Montbrun S (2007). TRIM5(alpha} Cytoplasmic Bodies Are Highly Dynamic Structures. Mol Biol Cell.

[ppat-0040016-b020] Short KM, Cox TC (2006). Subclassification of the RBCC/TRIM superfamily reveals a novel motif necessary for microtubule binding. J Biol Chem.

[ppat-0040016-b021] Buchner G, Montini E, Andolfi G, Quaderi N, Cainarca S (1999). MID2, a homologue of the Opitz syndrome gene MID1: similarities in subcellular localization and differences in expression during development. Hum Mol Genet.

[ppat-0040016-b022] Cainarca S, Messali S, Ballabio A, Meroni G (1999). Functional characterization of the Opitz syndrome gene product (midin): evidence for homodimerization and association with microtubules throughout the cell cycle. Hum Mol Genet.

[ppat-0040016-b023] Schweiger S, Foerster J, Lehmann T, Suckow V, Muller YA (1999). The Opitz syndrome gene product, MID1, associates with microtubules. Proc Natl Acad Sci U S A.

[ppat-0040016-b024] Wolf D, Goff SP (2007). TRIM28 mediates primer binding site-targeted silencing of murine leukemia virus in embryonic cells. Cell.

[ppat-0040016-b025] Tissot C, Mechti N (1995). Molecular cloning of a new interferon-induced factor that represses human immunodeficiency virus type 1 long terminal repeat expression. J Biol Chem.

[ppat-0040016-b026] Fridell RA, Harding LS, Bogerd HP, Cullen BR (1995). Identification of a novel human zinc finger protein that specifically interacts with the activation domain of lentiviral Tat proteins. Virology.

[ppat-0040016-b027] Gack MU, Shin YC, Joo CH, Urano T, Liang C (2007). TRIM25 RING-finger E3 ubiquitin ligase is essential for RIG-I-mediated antiviral activity. Nature.

[ppat-0040016-b028] Towers GJ (2005). Control of viral infectivity by tripartite motif proteins. Hum Gene Ther.

[ppat-0040016-b029] Bates P, Rong L, Varmus HE, Young JA, Crittenden LB (1998). Genetic mapping of the cloned subgroup A avian sarcoma and leukosis virus receptor gene to the TVA locus. J Virol.

[ppat-0040016-b030] Si Z, Vandegraaff N, O'Huigin C, Song B, Yuan W (2006). Evolution of a cytoplasmic tripartite motif (TRIM) protein in cows that restricts retroviral infection. Proc Natl Acad Sci U S A.

[ppat-0040016-b031] Perez-Caballero D, Hatziioannou T, Yang A, Cowan S, Bieniasz PD (2005). Human tripartite motif 5alpha domains responsible for retrovirus restriction activity and specificity. J Virol.

[ppat-0040016-b032] Sawyer SL, Wu LI, Emerman M, Malik HS (2005). Positive selection of primate TRIM5alpha identifies a critical species-specific retroviral restriction domain. Proc Natl Acad Sci U S A.

[ppat-0040016-b033] Sherer NM, Lehmann MJ, Jimenez-Soto LF, Ingmundson A, Horner SM (2003). Visualization of retroviral replication in living cells reveals budding into multivesicular bodies. Traffic.

[ppat-0040016-b034] Lehmann MJ, Sherer NM, Marks CB, Pypaert M, Mothes W (2005). Actin- and myosin-driven movement of viruses along filopodia precedes their entry into cells. J Cell Biol.

[ppat-0040016-b035] Lerner M, Corcoran M, Cepeda D, Nielsen ML, Zubarev R (2007). The RBCC gene RFP2 (Leu5) encodes a novel transmembrane E3 ubiquitin ligase involved in ERAD. Mol Biol Cell.

[ppat-0040016-b036] Nasioulas G, Hughes SH, Felber BK, Whitcomb JM (1995). Production of avian leukosis virus particles in mammalian cells can be mediated by the interaction of the human immunodeficiency virus protein Rev and the Rev-responsive element. Proc Natl Acad Sci U S A.

[ppat-0040016-b037] Sakuma R, Noser JA, Ohmine S, Ikeda Y (2007). Rhesus monkey TRIM5alpha restricts HIV-1 production through rapid degradation of viral Gag polyproteins. Nat Med.

[ppat-0040016-b038] Ishikawa H, Tachikawa H, Miura Y, Takahashi N (2006). TRIM11 binds to and destabilizes a key component of the activator-mediated cofactor complex (ARC105) through the ubiquitin-proteasome system. FEBS Lett.

[ppat-0040016-b039] Niikura T, Hashimoto Y, Tajima H, Ishizaka M, Yamagishi Y (2003). A tripartite motif protein TRIM11 binds and destabilizes Humanin, a neuroprotective peptide against Alzheimer's disease-relevant insults. Eur J Neurosci.

[ppat-0040016-b040] Sherer NM, Lehmann MJ, Jimenez-Soto LF, Horensavitz C, Pypaert M (2007). Retroviruses can establish filopodial bridges for efficient cell-to-cell transmission. Nat Cell Biol.

[ppat-0040016-b041] Mothes W, Boerger AL, Narayan S, Cunningham JM, Young JA (2000). Retroviral entry mediated by receptor priming and low pH triggering of an envelope glycoprotein. Cell.

[ppat-0040016-b042] Towers G, Bock M, Martin S, Takeuchi Y, Stoye JP (2000). A conserved mechanism of retrovirus restriction in mammals. Proc Natl Acad Sci U S A.

[ppat-0040016-b043] Saldanha AJ (2004). Java Treeview–extensible visualization of microarray data. Bioinformatics.

